# Quantitative trait association in parent offspring trios: Extension of case/pseudocontrol method and comparison of prospective and retrospective approaches

**DOI:** 10.1002/gepi.20243

**Published:** 2007-06-04

**Authors:** Eleanor Wheeler, Heather J Cordell

**Affiliations:** 1 The Wellcome Trust Sanger InstituteCambridge, UK; 2 Institute of Human Genetics, Newcastle UniversityUK

**Keywords:** family-based, regression, imprinting, TDT

## Abstract

The case/pseudocontrol method provides a convenient framework for family-based association analysis of case-parent trios, incorporating several previously proposed methods such as the transmission/disequilibrium test and log-linear modelling of parent-of-origin effects. The method allows genotype and haplotype analysis at an arbitrary number of linked and unlinked multiallelic loci, as well as modelling of more complex effects such as epistasis, parent-of-origin effects, maternal genotype and mother-child interaction effects, and gene-environment interactions. Here we extend the method for analysis of quantitative as opposed to dichotomous (e.g. disease) traits. The resulting method can be thought of as a retrospective approach, modelling genotype given trait value, in contrast to prospective approaches that model trait given genotype. Through simulations and analytical derivations, we examine the power and properties of our proposed approach, and compare it to several previously proposed single-locus methods for quantitative trait association analysis. We investigate the performance of the different methods when extended to allow analysis of haplotype, maternal genotype and parent-of-origin effects. With randomly ascertained families, with or without population stratification, the prospective approach (modeling trait value given genotype) is found to be generally most effective, although the retrospective approach has some advantages with regard to estimation and interpretability of parameter estimates when applied to selected samples. *Genet. Epidemiol*. 31:833, 2007. © 2007 Wiley-Liss, Inc.

## INTRODUCTION

Numerous methods have been proposed to test for association between a quantitative trait and a diallelic locus of interest. In a group of unrelated subjects, simple linear regression can be used to relate the quantitative trait phenotype to the genotype. However, this approach can be adversely affected by population stratification [Gauderman, 2003] and hence family-based designs are often preferred. Perhaps the simplest family-based design is to genotype a sample of unrelated phenotyped individuals and their parents, generating a set of parent-offspring trios. Analagous to the transmission/disequilibrium test (TDT) for disease traits [Spielman et al., 1993], tests that are robust to stratification can be derived by focussing on the transmission of the parental alleles to the offspring. If a given marker is not linked to a quantitative trait locus (QTL) (so that the marker alleles are transmitted randomly from parents to offspring), offspring quantitative phenotype is independent of offspring marker genotype given the parental marker genotypes [Whittaker et al., 2003]. This observation led Whittaker et al. [2003] and Gauderman [2003] to propose a test that is robust to population stratification, by adding terms that code for parental mating type into the linear regression equation. This approach (denoted QTDT_M_ by Gauderman [2003]) is closely related to tests previously proposed by Allison [1997] and Lunetta et al. [2000] (for details, see Gauderman [2003]).

An alternative approach was proposed by Fulker et al. [1999] and Abecasis et al. [2000]. These authors added terms to the linear regression model to separate out the within and between mating type information. The within mating type test was referred to as the hierarchical QTDT (HQTDT) by Gauderman [2003] and is the method implemented in the QTDT program [Abecasis et al., 2002]. Gauderman [2003] noted that for parent-offspring trios, HQTDT and QTDT_M_ are virtually identical with regards to inference about the effects of interest (genotype effects on the trait); however HQTDT has the advantage that it has also been extended to apply to general pedigrees [Abecasis et al., 2002]. Yang et al. [2000] described a similar model to the HQTDT. The differences between the models have little or no effect on the estimates and test of interest [Gauderman, 2003], and the Yang et al. [2000] method was therefore treated as equivalent to HQTDT by Gauderman [2003].

The above models are *prospective* models, that is, they model the quantitative phenotype in terms of the offspring genotype. They all assume that the quantitative traits are normally distributed, or else rely on the central limit theorem. To protect against the effects of possible deviations from either normality or selection on the trait, the HQTDT implemented in the QTDT program can carry out a permutation procedure based on permutation of genotypes to produce an empirical *P*-value. The connection between maximum likelihood inference under an assumed normal distribution and least-squares regression, however, means that in general we expect these regressionbased methods to be reasonably robust to small deviations of the trait from normality, even without use of permutation arguments.

An alternative approach is to use a *retrospective* approach, in which offspring genotype is modelled as a function of quantitative phenotype (possibly given parental genotypes). This approach is more akin to the original TDT of Spielman et al. [1993]. Such an approach provides the rationale for the family-based association test (FBAT) of Laird et al. [2000], although Lange et al. [2002] showed that this retrospective FBAT approach is in fact equivalent to implementing the HQTDT of Abecasis et al. [2000] via a score test rather than via a likelihood ratio test. Kistner and Weinberg [2004, 2005] describe a retrospective approach in which the offspring genotype is modelled as a function of their phenotype and parental genotypes, making no explicit assumptions about the distribution of the quantitative trait. This model, called the quantitative polytomous logistic (QPL) model, can be thought of as an extension of the log-linear model proposed by Weinberg et al. [1998] for qualitative traits.

The log-linear model for qualitative traits proposed by Weinberg et al. [1998] is very similar to the case/pseudocontrol approach for case-parent trios proposed by Cordell and Clayton [2002] (described in more detail by Cordell et al. [2004]). The main difference between the two approaches is that Cordell and Clayton [2002] model offspring genotypes conditional both on parental genotype and ascertainment through the affected offspring, whereas Weinberg et al. [1998] model the frequencies of the 15 possible trio types consisting of offspring genotype and parental mating type. The case/pseudocontrol approach can be thought of as a generalization of the TDT and the approaches of Schaid and Sommer [1993], Schaid [1996] and Weinberg et al. [1998], generalized to allow the fitting of more complex models where several linked and/or unlinked loci may contribute to disease via a combination of offspring and/or maternal genotype or haplotype effects, parent-of-origin effects and gene-gene or gene-environment interactions. Given the flexibility of the case/pseudocontrol approach, here we extend this approach to deal with quantitative traits, and compare the resulting method to original and extended versions of previously proposed quantitative trait association approaches.

## METHODS

### THE QTDT_M_

Gauderman [2003] and Whittaker et al. [2003] incorporate parental mating type as a fixed effect in a linear regression model. The model is of the form

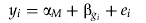
(1)
where *y*_*i*_ denotes a quantitative phenotype, *g*_*i*_ the genotype at a particular locus for the *i*th individual (*g*_*i*_ = 0, 1, 2 according to whether the genotype is 1/1, 1/2 (or 2/1) or 2/2), and α*M* (*M* = 1, …, 6) are mating-type specific intercepts. The residual *e*_*i*_ is assumed to be normally distributed with mean 0 and variance σ^2^. The formulation above suggests that the model is parameterized in terms of six mating-type parameters (the α_M_) and three child-genotype parameters (β_0_, β_1_ and β_2_), but, in fact, only two child-genotype parameters are estimable, with one of the genotype categories being chosen as the reference genotype category (the baseline genotype category to which the other genotype effects are compared). For example, if 1/1 is chosen as the reference genotype, β_0_ is set equal to 0 and the test of no association between *y* and *g* is *H*_0_:β_1_ = β_2_ = 0. Alternatively, if the heterozygous genotype 1/2 is chosen as the reference genotype, β_1_ is set equal to 0 and the test of no association between *y* and *g* is *H*0:β_0_ β_2_ = 0.

Similar to the HQTDT of Abecasis et al. [2000], the QTDT_M_ draws information from both within and between mating types. The HQTDT models the differences across mating types using a between mating type parameter whereas the QTDT_M_ uses the multiple fixed intercepts aM. Gauderman [2003] notes that inferences for the genotype effects are the same using the HQTDT and QTDT_M_ methods. The differences between the methods are in the estimates and interpretation of the mating-type specific intercepts, which are treated as random effects in the HQTDT and fixed effects in QTDT_M_.

### THE QPL METHOD

Kistner and Weinberg [2004, 2005] describe a retrospective model in which offspring genotype is modelled as a function of offspring phenotype and parental genotypes. The model is an extension of the log-linear model proposed by Weinberg et al. [1998] for qualitative traits and is fit using a polytomous logistic model with a generalized logit link function. Assuming parental mating symmetry in the population, there are six distinct parental mating types and the offspring genotype is modelled conditional on the offspring's quantitative trait (*y*_*i*_) and the parental mating type. Let *S*_*M*_ denote the set of possible offspring genotypes for mating type *M*. The summation 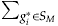 denotes the restricted summation over the offspring genotypes consistent with *S*_*M*_. The contribution of a trio to the likelihood is modelled as

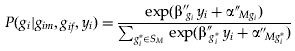
(2)
where 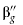 are parameters representing association between quantitative trait and genotype, and 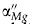 are nuisance parameters to account for non-Mendelianism and/or population stratification and depend on both parental mating type and the offspring genotype. In the formulation described by Kistner and Weinberg [2004], these parameters are denoted as β_*g*_ and α_*Mg*_, but here we instead use the double primed notation (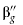 and 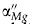) to distinguish these parameters from the parameters β_*g*_ and α_*M*_ used in the prospective formulation of equation (1). Kistner and Weinberg [2004] code the offspring, maternal and paternal genotypes as 0, 1 or 2 depending on the number of ‘variant’ alleles (here considered to be allele 2) they carry (the results of the test will be the same regardless which allele is considered to be the ‘variant’). For the *i*th trio, let the offspring's quantitative trait value be denoted by *y*_*i*_. Column 3 of Table I shows the conditional likelihoods for all combinations of parent and offspring genotypes that would result from equation (2) if all the parameters were estimable. Note that there are three child genotype parameters (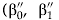 and 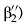) and seven nuisance parameters (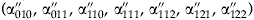) with 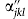 corresponding to the nuisance parameter for the category with (unordered) parental genotypes *j* and *k* and child genotype *l*. In practice, this model is overparameterized, and Kistner and Weinberg [2004] treat the heterozygous offspring genotype, 1 (1/2 or 2/1), as the reference genotype category. This results in a final model with six estimated parameters 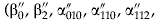, and 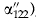, with 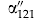 and 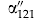 being set equal to zero, as shown in the fourth column of Table I. An alternative parameterization for the child's genotype parameters (which we shall we use in our simulation study) would be to set 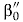 to zero and to estimate 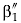 and 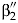. Regardless of the parameterization chosen, the categorization into three possible offspring genotype categories means that there are a maximum of three possible terms in the denominator of equation (2), clearly seen in the columns 3 and 4 of Table I.

**TABLE I:** The conditional likelihoods associated with the QPL model

		QPL likelihood	
		
Parents (*g_m_*,*g_f_*)	Offspring(g)	Overparameterized model	Six estimable parameters estimated
00	0	1	1
02	1	1	1
22	2	1	1
01	0	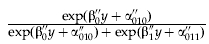	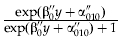
	1	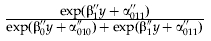	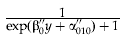
11	0	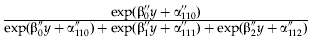	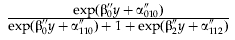
	1	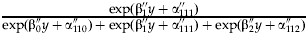	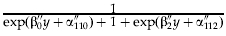
	2	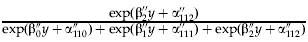	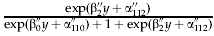
12	1	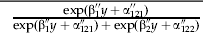	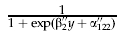

The likelihoods are proportional to *P*(*g*∣*g_m_*,*_f_*,*y*),corresponding to all combinations of (unordered) parent and offspring genotypes.

### THE QCPG METHOD

The method we propose is closely related to the approach by Kistner and Weinberg [2004, 2005], but the parameterization and implementation of the methods are somewhat different. Our approach derives from the case/pseudocontrol method for dichotomous traits [Cordell and Clayton, 2002, Cordell et al., 2004]. This method involves constructing (from a sample of case-parent trios) a sample of cases and matched pseudocontrols. We focus here on the ‘conditioning on parental genotypes’ (CPG) approach of Cordell et al. [2004], which generates pseudocontrols conditional on the mother's and father's genotypes (and possibly also conditional on some other event ξ, such as phase or parent-of-origin being determinable).

The extension of the CPG method to quantitative traits, here named the quantitative CPG (QCPG), is based on a calculation of the conditional likelihood of the offspring genotypes, conditional on the parental genotypes and the offspring phenotypes. For family *i*, let *g*_*i*_, *g*_*im*_, *g*_*if*_ be the offspring, maternal and paternal genotypes respectively, and let *y*_*i*_ be the offspring's quantitative trait. Then,

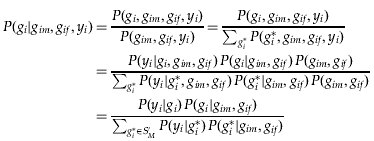

where 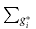 denotes summation over the four possible offspring genotypes and 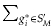 denotes summation over all possible offspring genotypes that could have been transmitted to the offspring given the parental genotypes (the probabilities 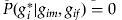 for offspring genotypes that are inconsistent with the parental genotypes). This is of the form of the case/pseudocontrol likelihood for qualitative traits [Cordell and Clayton, 2002] with the offspring's affection status replaced by a quantitative phenotype. The likelihood can be calculated via conditional logistic regression as implemented in standard statistical software. In Appendix A we show that the contribution of a trio to the likelihood may be assumed to be of the form

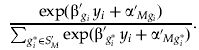

Here 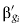 represent genotype effects, and 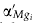 are nuisance parameters modelling non-Mendelianism and population stratification. The likelihood is similar to Kistner and Weinberg's QPL likelihood equation (2), except for the summation in the denominator. Columns 3 and 4 of Table II show the conditional probabilities corresponding to all combinations of parent and offspring genotypes for the QCPG method. By comparing column 4 of Tables I and II, and ignoring constants of proportionality, it can be seen that the QCPG likelihood is identical to the QPL likelihood, except for the offspring of two heterozygous parents. With two heterozygous parents, the sum in the denominator of the QCPG likelihood is over a maximum of four possible offspring genotypes. However, Kistner and Weinberg sum over a maximum of three possible offspring genotypes. For example, for a heterozygous offspring, the contribution to the QPL likelihood is

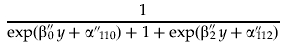

whereas for the QCPG method the contribution to the likelihood is

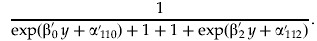

Essentially, in the QCPG formulation, we distinguish between the two possible heterozygote offspring genotypes 1/2 and 2/1 in the summation in the denominator (although in practice – assuming no parent-of-origin effects – the likelihood will be identical regardless of whether the observed offspring has genotype 1/2 or 2/1). As a result of the different likelihood formulations, interpretation of the nuisance parameters is different for the QCPG method and Kistner and Weinberg's QPL. The difference is most noticeable under the null with no population stratification or non-Mendelianism. In Appendix A we show that, for the QCPG method, the true values of the nuisance parameters α′ under the null with no population stratification or non-Mendelianism equal zero, whereas in Appendix B we show that for Kistner and Weinberg's QPL method, the true values of the nuisance parameters α″ are non-zero. Provided all six estimable parameters (two offspring genotype effects and four nuisance parameters) are freely estimated during likelihood maximization, inference for the parameters of interest (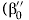 and 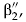, or 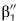 and 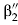, depending on which genotype category is chosen as reference) should not be affected by this result. However, the QPL result is slightly counter-intuitive, as one would generally expect that parameters that are specifically included in the likelihood to model certain effects (such as population stratification or non-Mendelianism) would take the value zero (i.e. be removable from the likelihood) when these ef fects do not, in fact, exist.

**TABLE II:** The conditional likelihoods associated with the QCPG model

		QCPG likelihood	
		
Parents (*g_m_*,*g_f_*)	Offspring (*g*)	Overparameterized model	Six estimable parameters estimated
00	0	1	1
02	1	1	1
22	2	1	1
01	0	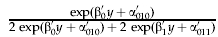	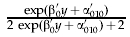
	1	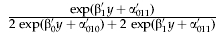	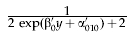
11	0	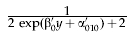	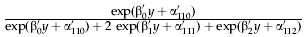
	1	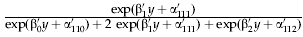	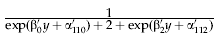
	2	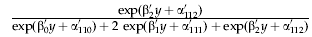	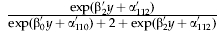
12	1	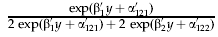	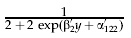
	2	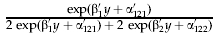	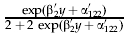

The likelihoods are proportional to *P*(*g*∣*g_m_*, *g_f_*, *y*), corresponding to all combinations of (unordered) parent and offspring genotypes.

An additional difference between the QCPG and QPL arises with regard to the number of nuisance parameters estimated (see Appendix C).

Liu et al. [2002] described a method that is closely related to the QCPG. Although the nuisance parameters are different, the likelihood is essentially of the same form. Rather than having six possible parental mating type parameters (α_*M*_), Liu et al. [2002] have a single baseline parameter, α, and number of additional parameters for the clusters of individuals whose trait differs from the population mean due to population stratification, δ_*i*_. However, without knowing the underlying population stratification, the δ_*i*_ parameters are unknown and cannot be estimated from the data. Liu et al. [2002] avoid having to estimate these nuisance parameters by showing that even in the presence of unobservable population stratification, it is still valid to test the null of no genetic effect via a score test, since population stratification has no effect on the null distribution of the test. Gauderman [2003] refers to the method of Liu et al. [2002] as the retrospective QTDT (RQTDT). To implement this method via a likelihood ratio test, Gauderman assumed that the quantitative trait follows a normal distribution with mean α + β_*gi*_ and variance σ_2_, without consideration of the δ_*i*_ parameters. In this implementation, a does not use information on the parental genotypes to model population stratification, although some information on the parental genotypes is still incorporated via the genotypes of the offspring and the pseudocontrols.

### EXTENSION OF QCPG TO MULTI-LOCUS HAPLOTYPES

Cordell et al. [2004] showed that the case/pseudocontrol approach can easily be extended to fit models for parent-of-origin effects, multiallelic markers, multiple linked loci in multiple unlinked regions, and gene-gene and gene-environment interactions, via an adjustment to the conditioning argument that results in differing numbers of pseudocontrols depending on the model being fitted. Here we extend this approach to quantitative traits. Consider models in which the genotype effects depend only on child's phased genotype. Define *g*_*i*_, *g*_*im*_, *g*_*f*_ as the offspring, maternal and paternal phase-known genotypes respectively, and *y*_*i*_ as the offspring's quantitative trait. The likelihood is very similar to that in equation (3) but we define an event χ as the event that the set of transmitted and untransmitted haplotypes from the parents can be deduced. The contribution to the conditional likelihood is

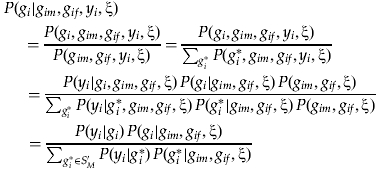

where 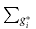 denotes summation over all possible offspring genotypes and 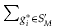 denotes summation over all possible offspring genotypes that could have been transmitted to the offspring given the parental genotypes. Under Mendelian inheritance the probabilities 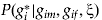 are equal for all 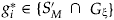 and equal zero otherwise, where Gχ denotes the set of offspring genotypes determined by χ. Then the contribution to the likelihood under Mendelian inheritance is given by

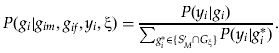

Note that, provided the models that are to be fitted do not depend on phase, one could also use the QCPG for analysis of unphased multilocus genotype data, in the same way that the CPG method can be used for unphased genotype data [Cordell et al., 2004].

### EXTENSIONS FOR MATERNAL GENOTYPE AND PARENT-OF-ORIGIN EFFECTS

Kistner et al. [2006] proposed an extension to the QPL approach to allow testing for maternally mediated effects and parent-of-origin effects. The likelihood factors into two parts. The first factor tests for genotype effects in the offspring and can be modelled using the original QPL method. The second factor tests for maternal genotype or parent-of-origin effects via a logistic regression model. Maternal genotype effects are incorporated by modelling the probability that the mother has more copies of the variant allele than the father for each mating type. Parent-of-origin effects are incorporated by additionally including a binary indicator variable, indicating whether the offspring inherited only one copy of the variant allele. This implies the child is heterozygous, and since the mother has more copies of the variant allele than the father, the variant allele must have been inherited from the mother.

We may also extend the QCPG method to allow for maternal genotype and parent-of-origin effects. The ‘conditioning on exchangeable parental genotypes’ (CEPG) method [Cordell et al., 2004] is an extension of the CPG approach to detecting parent-of-origin or maternal genotype effects by assuming exchangeability of parental genotypes. The method conditions on the set of parental genotypes but not on their order, generating additional pseudocontrols constructed by exchanging the genotypes of the mother and father. Here we extend this approach to quantitative traits. Maternal genotype effects are defined to be the direct effect of the maternal genotype on the offspring's quantitative trait, and parent-of-origin effects are defined (as in Weinberg et al. [1998]) to allow the offspring's quantitative trait to vary according to the parental origin of the variant allele, if present. Like Cordell et al. [2004], we introduce an additional conditioning event χ corresponding to the event that parent-of-origin and maternal genotype can be deduced in the trio. For quantitative trait CEPG method (denoted here QCEPG), the contribution of a trio to the QCEPG likelihood is

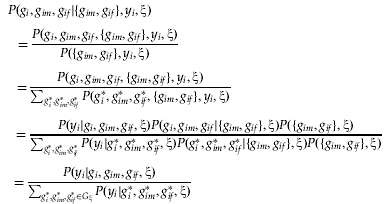

where {*g*_*im*_, *g*_*if*_} denotes the unordered set of parental genotypes and the final restricted sum is over the possible trios in which parent-of-origin (as well as maternal genotype) are deducible. Unlike Kistner and Weinberg's extension of the QPL method, the QCEPG method does not involve factoring the likelihood. In addition, the null hypothesis of no parent-of-origin effects considers the transmission of variant alleles from the mother to all offspring, not only those who are heterozygous. The contribution of the likelihood (see Appendix D) is assumed to be of the form

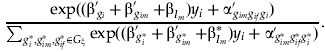

The offspring genotype effects are denoted by 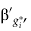, the maternal genotype effects by 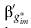 and parent-of-origin effects by 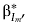, where *I*_*m*_ is an indicator of whether an offspring inherits a variant allele from the mother. The nuisance parameters 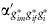 depend on the offspring genotype and the genotypes of the parents. For the QCPG, in the absence of maternal genotype or parent-of-origin effects, the nuisance parameters only depended on the parental mating type and the offspring genotype. However, in the QCEPG, the maternal genotype is of interest and needs to be included. No additional information is gained by incorporating the parent-of-origin indicator into the nuisance parameter since once the phaseknown genotypes of the parents and offspring are specified, and conditional on the fact that parent-oforigin can be deduced, parent-of-origin is also established. If only maternal genotype effects are of interest then the parent-of-origin indicator can simply be removed from the model and the nuisance parameters remain the same. However, if only parent-of-origin effects are of interest then the nuisance parameters are of the form 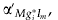, which depend on the parental mating type, the offspring genotype and the parent-of-origin indicator.

The QTDT_M_ method can also be extended to include maternal genotype effects, β_*gim*_, and parent-of-origin effects, β_*Im*_, through fitting the linear regression model

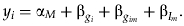

Trios in which parent-of-origin can be resolved can be found by first generating a case/pseudocontrol dataset as described in Cordell et al. [2004], specifying that the parental genotypes are exchangeable and parent-of-origin can be resolved. For the QTDT_M_ method, only the original offspring (the ‘case’) from the case/pseudocontrol dataset (together with information about maternal genotype and parent-of-origin status) is used in the prospective likelihood, whereas in the QCEPG method, the full set of cases and pseudocontrols is required for the retrospective likelihood.

## SIMULATION STUDY

### SINGLE-LOCUS SIMULATIONS

Simulations were performed to investigate the power and properties of the various methods described. Initially, a single diallelic QTL locus was considered. One thousand replicates of data were generated, each consisting of a number of genotyped trios (i.e. a single offspring with a quantitative trait and both parents). Bias in the resulting parameter estimates, 95% confidence intervals, power and type I error were examined. A method that performs well would be expected to give unbiased parameter estimation and to show approximately 95% confidence interval coverage. The importance of the nuisance parameters in the retrospective models was also investigated by examining the estimates obtained when they are removed from the model and also when the offspring genotype is used as a substitute.

For the single-locus model, six generating scenarios were considered as shown in Table 1 (**online**). Three different sampling schemes were employed: random sampling, one-tail sampling from the upper tail of the offspring trait distribution and two-tailed sampling from the upper and lower tails of the offspring trait distribution. Under random sampling, 500 parent-offspring trios were simulated per replicate where the offspring's quantitative trait was drawn from a normal distribution with genotype mean and standard deviation as shown in Table 1 (**online**). Population stratification was simulated by combining data in different proportions from two subpopulations, each of which was in Hardy-Weinberg equilibrium and showed random mating. The subpopulations had different allele frequencies and mean quantitative trait values, producing a spurious correlation between the quantitative trait and genotypes when the populations are combined. Under selected sampling from the extremes, 5,000 trios were generated per replicate, from which a subset were selected for analysis. For the two–tailed sampling scheme, 500 trios were selected from the 5,000 (i.e. the top and bottom 5% of the trait distribution). For the one-tail sampling scheme, we selected 1,000 trios from the 5,000 (i.e. the top 20%), as convergence problems were encountered when using only 500 trios under this sampling scheme.

Table III shows results for the first three scenarios with no population stratification and where the trios were randomly sampled. Under the null, all the methods gave unbiased estimates and reasonable confidence intervals, except for the QPL method where the nuisance parameters have been removed. This is expected since under the null, the α″ parameters in the QPL are nonzero and so their removal affects the resulting β″ estimates. Similarly, under the first alternative model (Alt 1) all the methods performed well except the QPL and QCPG methods where the nuisance parameters have been removed. The retrospective models in which the nuisance parameters have been replaced by the offspring genotype parameters give β′ and β″ estimates very close to the true means and reasonable coverage.

**TABLE III:** True and estimated means, standard deviations (SD) and coverage (CI) of the 95% confidence intervals for the single locus simulations with random selection and no population stratification

		Models											
		
		Null				Alt 1				Alt 2			
				
Method	Parameter	True mean	Mean	(SD)	CI	True mean	Mean	(SD)	CI	True mean	Mean	(SD)	(CI)
Linear regression	Constant	–	1.99	(0.46)	–	–	−0.01	(0.46)	–	–	−0.01	(0.46)	–
	β_1_	0.00	0.05	(0.05)	0.96	1.00	1.05	(0.50)	0.96	0.00	0.05	(0.50)	0.96
	β_2_	0.00	0.04	(0.48)	0.96	2.00	2.04	(0.48)	0.96	1.00	1.04	(0.48)	0.96
QTDT_M_	β_1_	0.00	0.08	(0.60)	0.95	1.00	1.08	(0.60)	0.95	0.00	0.08	(0.60)	0.95
	β_2_	0.00	0.07	(0.63)	0.95	2.00	2.07	(0.63)	0.95	1.00	1.07	(0.63)	0.95
	β_2_	0.00	0.07	(0.63)	0.95	2.00	2.07	(0.63)	0.95	1.00	1.07	(0.63)	0.95
QCPG	β^′^_1_	0.00	0.02	(0.16)	0.95	0.25	0.27	(0.17)	0.96	0.00	0.02	(0.16)	0.95
	β^′^_2_	0.00	0.02	(0.16)	0.95	0.50	0.51	(0.17)	0.97	0.25	0.25	(0.16)	0.96
QPL	β^″^_1_	0.00	0.02	(0.16)	0.95	0.25	0.27	(0.17)	0.97	0.25	0.25	(0.16)	0.96
	β^″^_1_	0.00	0.02	(0.16)	0.95	0.50	0.51	(0.17)	0.97	0.25	0.25	(0.16)	0.96
QCPG αs removed	β^″^_1_	0.00	0.02	(0.11)	0.96	0.25	0.26	(0.15)	0.96	0.00	0.02	(0.14)	0.96
	β^″^_1_	0.00	0.01	(0.11)	0.95	0.50	0.40	(0.16)	088	0.25	0.23	(0.15)	0.96
QPL α^″^s removed	β^″^_1_	0.00	0.10	(0.10)	0.87	0.25	0.27	0.14	0.97	0.00	0.00	(0.13)	0.97
	β^″^_2_	0.00	0.01	(0.11)	0.95	0.50	0040	(0.16)	0.88	0.25	0.23	(0.15)	0.96
QCPG α^′^s replaced by	β ^′^_1_	0.00	0.02	(0.15)	0.96	0.25	0.27	(0.14)	0.88	0.25	0.21	(0.13)	00.97
offspring genotyped *g*	β^′^_2_	0.00	0.07	(0.10)	0.92	0.50	0.40	(0.14)	0.88	0.25	0.21	(0.13)	0.97
QPL α^″^s replaced by	β^″^_1_	0.00	0.02	(0.15)	0.96	0.25	0.24	(0.16)	0.97	0.00	0.00	(0.15)	0.96
offspring genotype (*g*)	β^″^_2_	0.00	0.01	(0.15)	0.96	0.50	0.47	(0.16)	0.96	0.25	0.23	(0.15)	0.96

The simulation parameters are as shown in Table 1 (online supplementary materials).

The results for the three scenarios in the presence of population stratification and under random sampling are shown in Table IV. Simple linear regression showed the expected bias in the estimates of β and poor coverage of the estimated 95% confidence intervals since the population stratification is not accounted for in the method. Under the first null model, as in the case without population stratification (Table III), the QPL method with the β″ parameters removed did not perform well (coverages 0.87 and 0.92 instead of 0.95). The remainder of the methods perform well under both null models, even under population stratification. Substitution of the four nuisance parameters by the offspring genotype in the retrospective methods appears to account sufficiently for the population stratification. Under the alternative with population stratification, only the prospective QTDT_M_ method produced unbiased estimates and correct coverage. The retrospective methods (QCPG and QPL) produced biased estimates, as expected (see Appendices A and C).

**TABLE IV:** True and estimated means, standard deviations (SD) and coverage (CI) of the 95% confidence intervals for the single locus simulations with random selection and with population stratification

		Models											
		
		Null				Alt 1				Alt 2			
				
Method	Parameter	True mean	Mean	(SD)	CI	True mean	Mean	(SD)	CI	True mean	Mean	(SD)	CI
Linear regression	Constant	–	2.29	(0.21)	–	–	0.02	(0.10)	–	–	1.84	(0.19)	–
	β_1_	0.00	−2.06	(0.22)	0.00	0.00	0.25	(0.15)	0.76	1.00	0.15	(0.24)	0.07
	β_2_	0.00	−2.06	(0.22)	0.00	0.00	1.31	(0.14)	0.00	2.00	0.34	(0.20)	0.00
QTDT_m_	β_2_	0.00	0.01	(0.33)	0.95	0.00	0.00	(0.17)	0.98	1.00	1.01	(0.31)	0.95
	β_2_	0.00	0.01	(0.39)	0.94	0.00	0.00	(0.24)	0.97	2.00	2.01	(0.35)	0.94
QCPG	β^′^_1_	0.00	0.01	(0.20)	0.95	0.00	0.01	(0.15)	0.95	0.25	0.68	(0.24)	0.61
	β^′^_2_	0.00	0.00	(0.21)	0.95	0.00	0.00	0.16	0.96	0.50	1.23	(0.27)	0.17
QPL	β^″^_1_	0.00	0.01	(0.20)	0.95	0.00	0.01	(0.15)	0.95	0.25	0.68	(0.24)	0.61
	β^″^_2_	0.00	0.00	(0.21)	0.95	0.00	0.00	(0.16)	0.96	0.50	1.23	(0.27)	0.17
QCPG α^′^s removed	β^′^_1_	0.00	0.00	(0.10)	0.96	0.00	0.01	(0.14)	0.95	0.25	0.14	(0.09)	0.81
	β^′^_2_	0.00	0.00	(0.12)	0.95	0.00	0.00	(0.16)	0.96	0.50	0.29	(0.10)	0.50
QPL α^″^s removed	β^″^_1_	0.00	0.08	(0.09)	0.91	0.00	0.02	(0.14)	0.96	0.25	0.20	(0.09)	0.92
	β^″^	0.00	0.01	(0.11)	0.97	0.00	0.01	(0.14)	0.96	0.50	0.30	(0.09)	0.50
QCPG α^′^s replaced by	β^′^_1_	0.00	0.00	(0.19)	0.96	0.00	0.01	(0.14)	0.95	0.25	0.62	(0.22)	0.66
offspring genotype(g)	β^′^_2_	0.00	−0.01	(0.20)	0.96	0.00	0.00	(0.16)	0.95	0.50	1.11	(0.23)	0.25
QPL α^″^s replaced by	β^″^_1_	0.00	−0.03	(0.19)	0.95	0.00	0.01	(0.14)	0.95	0.25	0.56	(0.22)	0.79
offspring genotype (g)	β^″^_2_	0.00	−0.07	(0.19)	0.96	0.00	0.04	(0.15)	0.95	0.50	1.00	(0.22)	0.46

The simulation parameters are as shown in Table 1 (**online**).

Parameter estimates under one-tail selected sampling are shown in online Tables 2 and 3 (**online**). The results under the null (both with and without population stratification) are the same as those found in the unselected case. Under the alternative with no population stratification, both prospective models (simple linear regression and QTDT_M_) show biased estimates and incorrect coverage of the 95% confidence intervals. This is because the methods cannot account for the selection on quantitative trait value. By conditioning on the trait values, the retrospective models should be robust to selection. However, Table 2 (**online**) suggests that these methods are producing biased estimates. By looking at the median genotype effect estimates (data not shown), we found that the bias is due to a small number of outlying observations. The medians for the QCPG and QPL methods (with the true nuisance parameters and with the nuisance parameters replaced by the offspring genotype) are very close to the true means. Under the alternative with population stratification, all methods performed poorly, producing biased estimates and incorrect coverage. Here, the prospective model QTDT_M_ fails since it cannot account for selection on quantitative trait value and the retrospective models, QCPG and QPL, fail to estimate the nuisance parameters under the alternative with population stratification. Similar results were observed using the two-tailed sampling scheme (Tables 4 and 5 (**online**)). Without population stratification (Table 5 (**online**)), it can be seen that the bias in the estimates using simple linear regression is not as great under two-tailed sampling as found when sampling only from the upper tail of the trait distribution (note that under the alternative, the assumption of homoscedasticity of the residuals is violated under the one-tailed sampling scheme).

Powers/type I errors are shown in Table 6 (**online**). Since the powers to achieve *P* value of 0.001 for the different methods are all 1.0 under two-tailed sampling, we also investigated the power to achieve a more stringent significance level in this case. Under the null with no population stratification, removal of the nonzero nuisance parameters in the QPL method generates a bias in the estimates and hence increased type I error rates, most clearly seen in Table 6 (**online**) for the random and one-tail sampling schemes. The remaining methods all have type I error rates close to or less than the critical values. Highest power to detect a genotypic effect is seen with the linear regression method for the random and two-tailed sampling schemes and with the QCPG method with the α′ parameters removed for the one-tail sampling scheme (powers are meaningless for the QPL method with no α″ parameters since the type I errors are incorrect). In all cases, the highest powers to detect a genotypic effect are seen for the two-tailed selected sampling scheme, selecting from the upper and lower tails of the offspring trait distribution. In contrast, selection from only the upper tail of the offspring trait distribution actually decreases the power to detect a genetic effect compared to the random sampling scheme, despite having the largest sample size, except for the QCPG method with the α′ parameters removed. These results also show that, although under the alternative with selected sampling the QTDT_M_ method showed biased estimates and poor coverage of the 95% confidence intervals, the method can still be used to test for a genetic effect, as the type 1 error is correct. In fact, the large bias in the estimates seen when using a two-tailed sampling scheme actually increases the power to detect an effect compared to random sampling, although this power increase may also be due to the fact that the selected subjects carry more information, since they are concentrated at the extremes of the trait distribution.

Under population stratification, Table 6 (**online**) shows that for all sampling schemes the simple linear regression method has increased type I error rates. Since linear regression cannot account for the population substructure, the resulting bias in the estimates generates a large number of false-positive associations. The QPL method with the α″ parameters removed also has type I errors larger than the nominal values, particularly when selecting from the upper tail of the offspring trait distribution, as found in the case of no population stratification. The type I errors for the QTDT_M_ method under population stratification for the first null model are slightly larger than expected, across all three sampling schemes. Similarly, the type I error is inflated for the second null model under the two-tailed sampling scheme. The remainder of the methods have type I errors close to the nominal values. Therefore, the retrospective methods (QCPG and QPL) with the correct nuisance parameters or with the nuisance parameters replaced by the offspring genotype can be used (and indeed have high power) to test for a genetic effect, even under circumstances where they produce biased estimates.

### MULTI-LOCUS HAPLOTYPES

Simulations were carried out to investigate the effect of the nuisance parameters (intended to account for population stratification) when the methods are extended to multi-locus haplotypes. Note that, as originally proposed, the QTDT_M_ method (and simple linear regression) only apply to single loci: to extend these methods to multi-locus haplotypes it is necessary to first infer the child's (and if necessary, the parents') haplotypes given the observed genotype data, as is done in the first stage of the CPG and QCPG methods [Cordell et al., 2004]. The resulting haplotype variables may then be entered as predictor variables into equation (1).

Tables V and VI show the results of simulations in which the offspring quantitative trait was influenced by genotype at two linked diallelic markers assumed to be in moderate LD. The four possible haplotypes, 1-1, 1-2, 2-1 and 2-2, had haplotype frequencies and haplotype means as shown in Table 7 (**online**). Additive effects of haplotypes were assumed so that for each trio, the offspring's quantitative trait was drawn from a normal distribution whose mean was the sum of the two haplotype means. For each simulation, 1,000 replicates of data were generated, each replicate consisting of 1,000 parent-offspring trios with random selection or 1,000 trios selected from 10,000 in either one-tail (top 10%) or two-tailed (top and bottom 5%) sampling from the extremes of the offspring trait distribution.

**TABLE V:** Mean estimates and standard deviations of simulated two-locus haplotype effects using the simple linear regression, QTDT_M_ (with different sets of nuisance parameters) and the QCPG (with different sets of nuisance parameters)

		Null model							Alternative model						
			
Sampling scheme No.of replicates			Random 973		Top 980		Top+bottom			Random 978		TOP 132		Top+bottom	
									
Method	Parameter	True mean	Mean	(SD)	Mean	(SD)	Mean	(SD)	True mean	Mean	(SD)	Mean	(SD)	Mean	(SD)
Linear regression	β_2_	0.00	0.00	(0.08)	0.00	(0.03)	−0.01	(0.17)	1.00	1.00	(0.08)	0.21	(0.06)	2.18	(0.19)
	β_3_	0.00	0.00	(0.07)	0.00	(0.03)	0.00	(0.15)	2.00	2.00	(0.07)	0.63	(0.05)	3.52	(0.05)
	β_4_	0.00	0.00	(0.07)	0.00	(0.03)	0.00	(0.14)	3.00	3.00	(0.07)	1.17	(0.06)	4.08	(0.08)
QTDT_m_ all αs	β_2_	0.00	0.00	(0.11)	0.00	(0.05)	0.00	(0.27)	1.00	1.00	(0.11)	0.16	(0.15)	1.70	(0.28)
	β_3_	0.00	0.00	(0.11)	0.00	(0.04)	0.00	0.22	2.00	2.00	(0.10)	0.59	(0.12)	3.52	(0.14)
	β_4_	0.00	0.00	(0.10)	0.00	(0.04)	0.00	(0.20)	3.00	3.00	(0.10)	1.08	(0.16)	4.50	(0.14)
QTDT_m_ αs replaced by	β_2_	0.00	0.00	(0.11)	0.00	(0.05)	0.00	(0.26)	1.00	1.00	(0.11)	0.15	(0.14)	2.21	(0.22)
parental genotypes	β_3_	0.00	0.00	(0.10)	0.00	(0.04)	0.00	(0.22)	2.00	2.00	(0.10)	0.57	(0.12)	3.56	(0.08)
	β_4_	0.00	0.00	(0.10)	0.00	(0.04)	0.00	(0.119)	3.00	3.00	(0.10)	1.10	(0.14)	4.12	(0.06)
QTDT_m_ αs at both loci	β_2_	0.00	0.00	(0.10)	0.00	(0.04)	0.00	(0.22)	1.00	1.00	(0.10)	0.14	(0.10)	1.79	(0.21)
	β_3_	0.00	−0.01	(0.09)	0.00	(0.04)	0.00	(0.19)	2.00	1.99	(0.09)	0.60	(0.10)	3.78	(0.10)
	β_4_	0.00	0.00	(0.09)	0.00	(0.04)	0.00	(0.18)	3.00	3.00	(0.09)	1.12	(0.13)	4.34	(0.10)
QCPG all α^′^s	β^′^_2_	0.00	0.00	0.13	0.01	(0.31)	0.00	(0.06)	1.00	1.11	(0.18)	0.68	(0.90)	1.36	(0.96)
	β^′^_3_	0.00	−0.01	(0.11)	−0.01	(0.25)	0.00	(0.05)	2.00	2.23	(0.22)	1.76	(0.83)	2.69	(1.43)
	β^′^_4_	0.00	0.00	(0.11)	0.00	(0.25)	0.00	(0.05)	3.00	3.44	(0.36)	2.80	(0.97)	3.83	(1.56)
QCPG α^′^s replaced by	β^′^_2_	0.00	0.00	(0.12)	0.00	(0.28)	0.00	(0.06)	1.00	1.06	(0.14)	0.86	(0.76)	1.32	(0.88)
offspring genotype (g)	β^′^_3_	0.00	0.00	(0.10)	0.00	(0.24)	0.00	(0.05)	2.00	2.12	(0.19)	1.86	(0.67)	2.60	(1.10)
	β^′^_4_	0.00	0.00	(0.10)	0.00	(0.23)	0.00	(0.04)	3.00	3.19	(0.27)	2.87	(0.76)	3.67	(1.17)

Simulated with no population stratification

**TABLE VI:** Mean estimates and standard deviations of simulated two-locus haplotype effects using the simple linear regression, QTDTM (with different sets of nuisance parameters) and the QCPG (with different sets of nuisance parameters)

		Null model							Alternative model						
			
			Random 995		Top 941		Top+bottom 995			Random 996		Top 82		Top+bottom 502	
									
Method	Parameter	True mean	Mean	(SD)	Mean	(SD)	Mean	(SD)	True mean	Mean	(SD)	Mean	(SD)	Mean	(SD)
Linear regression	β_2_	0.00	3.30	(0.28)	0.00	(0.04)	4.14	(0.39)	1.00	4.30	(0.28)	0.26	(0.14)	0.96	(0.32)
	β_3_	0.00	2.07	(0.37)	0.00	(0.05)	2.06	(0.41)	2.00	4.07	(0.37)	0.31	(0.10)	6.74	(0.64)
	β_4_	0.00	4.07	(0.16)	0.00	(0.03)	6.22	(0.17)	3.00	7.07	(0.15)	0.50	(0.09)	9.21	(0.02)
QTDT_m_ allαs	β_2_	0.00	−0.01	(0.42)	0.00	(0.06)	0.00	(0.51)	1.00	0.99	(0.42)	0.23	(0.19)	0.90	(0.45)
	β_3_	0.00	−0.01	(0.42)	0.00	(0.07)	0.00	(0.44)	2.00	1.99	(0.42)	0.30	(0.14)	5.73	(0.93)
	β_4_	0.00	−0.01	(0.33)	0.00	(0.04)	0.00	(0.41)	3.00	2.99	(0.33)	0.50	(0.13)	8.06	(0.50)
QTDT_m_ αs replaced by	β_2_	0.00	−0.01	(0.43)	0.00	0.06	−0.01	(0.51)	1.00	0.99	(0.43)	0.13	(0.23)	0.84	(0.39)
parental genotypes	β_3_	0.00	−0.01	(0.44)	0.00	0.06	0.00	(0.45)	2.00	1.99	(0.44)	0.17	(0.23)	6.48	(0.74)
	β_4_	0.00	−0.01	(0.33)	0.00	0.04	0.00	(0.42)	3.00	2.99	(0.33)	0.37	(0.23)	8.79	(0.16)
QTDT_m_αs at both loci	β_2_	0.00	0.20	(0.37)	0.00	(0.05)	−0.01	(0.47)	1.00	1.20	(0.37)	0.24	(0.16)	0.66	(0.36)
	β_3_	0.00	0.23	(0.35)	0.00	(0.05)	0.00	(0.41)	2.00	2.23	(0.35)	0.31	(0.11)	6.45	(0.73)
	β_4_	0.00	0.04	(0.32)	0.00	(0.04)	0.00	(0.41)	3.00	3.03	(0.32)	0.51	(0.10)	8.53	(0.26)
QCPG all α^′^s	β^′^_2_	0.00	0.00	(0.05)	0.01	(0.32)	0.00	(0.04)	1.00	−0.03	(0.08)	8.83	(12.9)	1.07	(0.55)
	β^′^_3_	0.00	0.00	(0.05)	0.00	(0.36)	0.00	(0.03)	2.00	0.20	(0.09)	9.59	(12.7)	3.30	(1.73)
	β^′^_4_	0.00	0.00	(0.03)	0.00	(0.23)	0.00	(0.02)	3.00	0.44	(0.10)	10.51	(12.7)	4.13	(1.77)
QCPG α^′^s replaced by	β^′^_2_	0.00	0.00	(0.03)	0.01	(0.29)	0.00	(0.02)	1.00	0.08	(0.04)	7.64	(9.15)	1.05	(0.50)
offspring genotype (g)	β^′^_3_	0.00	0.00	(0.03)	0.01	(0.32)	0.00	(0.02)	2.00	0.14	(0.04)	8.41	(9.01)	3.46	(1.85)
	β^′^_4_	0.00	0.00	(0.03)	0.00	(0.22)	0.00	(0.02)	3.00	0.31	(0.05)	9.31	(9.03)	4.30	(1.92)

Simulated with population stratification

Three methods were considered, simple linear regression, QTDT_M_ and the retrospective method QCPG. The QPL method was not considered as it is so closely related to the QCPG. Simple linear regression does not have any additional parameters to account for population stratification. The QTDT_M_ and QCPG methods, however, have a significant number of nuisance parameters when the methods are extended to multi-locus haplotypes. For example, for QTDT_M_, the number of possible mating types (assuming parental mating symmetry) is 55, a large increase from the 6 in the single-locus case. Therefore, in addition to considering the models in which the ‘correct’ nuisance parameters are used, we considered models in which the number of nuisance parameters were reduced. For the QTDT_M_ we considered either including in the model the single-locus mating-type parameters for each locus, or including maternal and paternal genotype (rather than mating-type) parameters. For the QCPG method, replacing the nuisance parameters by the offspring genotype (g) was considered.

Table V shows the results for the case with no population stratification. Also shown is the number of replicates that converged from the original 1,000. Convergence problems were probably a small sample size problem, due to the large numbers of parameters to estimate in the models. Under the null, all the methods produced unbiased parameter estimates, regardless of selection scheme. Under the alternative with no selection, the prospective methods (simple linear regression and QTDT_M_ with the different sets of α parameters) gave unbiased parameter estimates. The retrospective QCPG method shows some small-sample bias in the estimates when the full set of ‘correct’ α′ parameters was used and similar bias when the α′ parameters were replaced by the offspring genotype *g*. This bias disappeared when 10,000 trios (as opposed to 1,000) were used (data not shown). Under the alternative with selection, only the retrospective QCPG method when all the ‘correct’ α′ parameters are used, or when the α′ are replaced by the offspring genotype, gave estimates close to the true mean.

The sensitivity of the estimates to the way the nuisance parameters are modelled is most pronounced under population stratification as shown in Table VI. Under the null with random selection, the simple linear regression method produces biased estimates, as does the QTDT_M_ method in which the correct α's are replaced by those that would be generated by considering the loci individually. The remaining methods, QTDT_M_ with the full set of α parameters or with parental genotype parameters, and the QCPG methods with the different sets of nuisance parameters, all have unbiased estimates. Under the null with selection from the upper tail of the offspring trait distribution, all of the methods produced unbiased parameter estimates. For the two-tailed sampling scheme, linear regression showed the expected bias in parameter estimates but QTDT_M_ with the correct α's, QTDT_M_ with parental genotypes and the retrospective methods (with the different sets of α′ parameters) produced unbiased parameter estimates. Under the alternative with random selection only the prospective QTDT_M_ method (with the different sets of α's) produced unbiased estimates: as explained in Appendices A and C, the nuisance parameters for the QPL and QCPG will not be correctly estimated under population stratification, except under the null. Under the alternative with selection, all the methods gave biased estimates as found in the single-locus case.

We also investigated the QTDT_M_ and QCPG methods with a single replicate of data generated under a three-locus haplotype model (data not shown). Results were broadly similar to the twolocus haplotype results, except that the QCPG method required a very large number (50,000) trios to produce unbiased estimates, while QTDT_M_ generally achieved convergence and unbiased parameter estimation with only 1,000 trios.

### STEPWISE PROCEDURE

A stepwise procedure (results not shown), as used by Cordell et al. [2004] for disease traits, was used to compare the prospective QTDT_M_ (using the full set of nuisance parameters) with the QCPG method with the ‘correct’ α′ parameters replaced by the offspring genotype, under models with and without population stratification and selection. In general, the pattern of results in terms of power and type 1 error was as expected, with the QTDT_M_ method being the more powerful in general. Under population stratification we found that the Type I errors were slightly too large for the QTDT_M_ method under random sampling, consistent with the results observed (Table 6 (**online**)) in the single locus simulations. Additional simulations (data not shown) indicated that this problem could be solved by use of Wald tests incorporating robust ‘information sandwich’ variance estimates [Huber, 1967], rather than likelihood ratio tests or Wald tests with the usual variance estimate (which equals minus the inverse of the Hessian matrix). We also investigated the power and type 1 error of the stepwise approach when applied to non-normally distributed traits and found that both QTDT_M_ and QCPG appear to be suitable for the analysis of traits that deviate slightly from normality. Neither method was found to be suitable for the analysis of very nonnormally distributed traits, although it is worth noting that the prospective QTDT_M_ method could easily be extended to enable the analysis of nonnormal traits by use of robust regression, a generalised linear model (GLM), or by assuming a variance-mean relationship, according to the departure from normality.

### MATERNAL GENOTYPE AND PARENT–OF–ORIGIN EFFECTS

The previous single-locus simulations were modified to include maternal genotype and parent-oforigin effects. For each replicate, 1,000 trios were generated in which the offspring's quantitative trait was influenced by its own genotype, and by either the mother's genotype, or whether the offspring received a variant allele from the mother, or both. Under the alternative, 100 replicates of data were generated. Under the null (no maternal genotype effects or no parent-of-origin effects) 1,000 replicates of data were generated. The QCEPG and QTDT_M_ methods were implemented in Stata. For Kistner and Weinberg's approach [Kistner et al., 2006], the SAS macro provided at http://dir.niehs.nih.gov/dirbb/weinbergfiles/qpl.htm was used. The expected effect estimates for QCEPG and QTDT_M_ should be the same, since the traits were simulated to have unit variance. The offspring reference category was chosen to be the 1/1 genotype, and β1 and β2 are the estimated effects for the 1/2 (2/1) and 2/2 genotypes respectively. Similarly, maternal genotype effects are denoted as β_*m1*_ and β_*m2*_, and parent-of-origin effects by β_*I*_. However, the expected estimates for Kistner and Weinberg's method should be slightly different. In their method the reference category for the offspring genotype effects is the heterozygous genotype, rather than the homozygous (1/1) genotype. The maternal effects, denoted by δ_01_ and δ_12_, compare the difference in quantitative trait for a mother with 1 variant allele to a mother with 0 variant alleles, and a mother with 2 variant alleles to a mother with 1 variant allele respectively (while in the QCEPG method, both comparisons are made with the homozygous 1/1 genotype category). The estimates for the parent-of-origin effects (λ1) in the QPL represent the log odds that a heterozygous child inherits a maternal copy of the variant allele instead of a paternal copy, per unit increase in trait value. Although based only on heterozygous offspring, these parameters are expected be the same as for the QCEPG and QTDT_M_ methods.

Tables 8 and 9 (**online**) show the true effects, the estimated means and standard deviations. All three methods produce reasonable estimates under the null. The results for the prospective QTDT_M_ method show the least bias. Under the alternative, the retrospective methods show a bias when parent-of-origin effects are present. The QPL appears to produce parent-of-origin effects of approximately 0.5, when they would have been expected to be 1. This may be due to some unrecognised difference in the parameterizations: the QCEPG uses the original parent-of-origin parameterization of Weinberg et al. [1998], whereas the QPL uses a parameterization closer to the alternative parameterization suggested by Weinberg [1999]. Table 10 (**online**) shows the powers and type I errors. The type I errors for the QPL and QTDT_M_ methods seem reasonable. However, the type I error for the QCEPG method when testing maternal genotype effects is very large, although this appears to be a small-sample issue as it improved in simulations with a larger number of trios (data not shown). Overall, the extension of the QPL method had the highest power to detect either a maternal genotype or parent-of-origin effect.

## DISCUSSION

In this paper, we have extended the case/pseudocontrol association approach for dichotomous phenotypes [Cordell and Clayton, 2002] to perform association analysis with quantitative traits. This approach is very similar to the QPL approach proposed by Kistner and Weinberg [2004], but uses a slightly more intuitive parameterization and extends more naturally to allow analysis of multiallelic markers, multiple linked loci, multiple unlinked regions, parent-of-origin or maternal genotype effects, gene-gene and gene-environment interactions, using the same formulation as Cordell et al. [2004]. We compared this approach to a prospective aproach, the QTDT_M_ and also extended the QTDT_M_ to allow analysis of multiple linked loci (including multi-locus haplotypes), parent-of-origin or maternal genotype effects. Other extensions to the QTDT_M_ follow naturally.

All the methods incorporate nuisance parameters intended to account for population stratification. When considering multi-locus haplotypes, the number of nuisance parameters can dramatically increase, and so it is important to find ways to reduce the number of nuisance parameters. It was found that replacing the nuisance parameters by the offspring genotype in the retrospective methods worked almost as well as the full model, and replacing the nuisance parameters by parental genotypes worked well for the QTDT_M_. In our simulations, it was assumed that both parents came from the same sub-population. If, in fact, matings occurred between individuals from different sub-populations, one might not expect these approximations to work as well as fitting the full set of nuisance parameters.

Although the retrospective approaches had some advantages with regard to estimation of parameters under selected sampling, in general we found the prospective QTDT_M_ to be the most efficient approach, requiring smaller sample sizes to achieve convergence and asyptotic behaviour. In addition, the parameter estimates provided by the QTDT_M_ have a more intuitive interpretation, corresponding to the direct genotype effects on the trait, whereas the retrospective approaches estimate parameters that are scaled by division by the unknown (although potentially estimable) trait variance. Covariates are also more easily incorporated into the QTDT_M_ framework, simply by adding them in as terms in the regression equation, although it would be possible to incorporate covariates in the retrospective approaches, either by first regressing the traits on covariates of interest and performing subsequent analysis on the residuals, or by using methods such as those described by Lim et al. [2005].

The QTDT_M_ method was found to be the only method suitable for estimation of effects under the alternative hypothesis with population stratification (assuming random sampling). Under population stratification, it was necessary to use robust ‘information sandwich’ variance estimates to achieve correct type 1 errors and confidence interval coverage with the QTDT_M_. This is possibly because the parental mating-type stratification parameters act as a surrogate for population membership in the sense that they soak up the mean level of bias induced by population stratification, but do they not fully account for population membership, so that the distribution of trait within parental mating-type classes violates the assumption of normality, even if this asssumption holds within each sub-population.

The analyses described here assumed availability of a dataset consisting of parent-offspring trios, with no missing genotype data. A natural extension of the methods proposed here would be to consider analysis of large extended pedigrees and/or missing genotype data. The QPL has previously been extended to allow analysis of multiple siblings and missing parents [Kistner and Weinberg, 2005] while an approach asymptotically equivalent to QTDT_M_, namely the HQTDT of Abecasis et al. [2000], has been extended to apply to pedigrees of arbitrary structure [Abecasis et al., 2002]. However, these approaches focus on testing rather than estimation of effects and apply only to a single locus at a time. A natural way to extend the QCEPG and QTDT_M_ approaches developed here for analysis of general pedigrees would be to perform tests using Wald tests and incorporate robust ‘information sandwich’ variance estimates that cluster observations according to pedigree [Huber, 1967]. An alternative approach would be to use a random-effects modelling framework [Xu and Shete, 2006]. With regards to missing genotype data, methods that sample or average over the possible genotype configurations consistent with the observed genotype data, in the correct proportions [Cordell, 2006], could be considered. Investigation of these approaches and their behaviour under complex disease models, in the presence of population stratification, will form the basis of future work.

## APPENDIX A

### EXPRESSION OF QCPG LIKELIHOOD

The retrospective QCPG likelihood is parameterized in terms of parameters of interest (offspring genotype effects, denoted β′) and several nuisance parameters (denoted α′) as shown in Table II. Here we express the β′ and α′ parameters in the QCPG likelihood in terms of various parameters (denoted α and β) in a prospective model for trait given genotype. We do not propose to reparameterize the QCPG likelihood of Table II in terms of these prospective parameters before maximization. Rather, we continue to freely estimate the β′ and α′ parameters when we maximise the QCPG likelihood. However, we use the relationship between the retrospective and prospective parameters to inform our understanding and interpretation of the restrospective parameters, since the α and β parameters in the prospective model are generally more intuitively interpretable than those on the retrospective scale.

For a normally distributed quantitative trait *Y* with mean μ and variance σ^2^, the probability distribution function of the observed trait *y*_*i*_ is given by

(7)

For a trio with parental mating type *M* and offspring genotype *g*_*i*_, μ equals the mean of the prospective QTDT_M_ approach from equation (1), that is μ = α*M* + β_*gi*_. Equation (7) becomes

(8)

From equation (3), the contribution of a trio to the likelihood can be expressed as

(9)

So, from equations (8) and (9), the contribution of a trio to the likelihood for a normally distributed trait with mean α*M* + β_*gi*_ and variance σ^2^ is given by

(10)

In each offspring's contribution to the likelihood, the quantitative trait, *y*_*i*_, its variance, σ^2^, and the parental mating type parameter α*M* are the same for the offspring (the ‘case’) and the pseudocontrols. Therefore, cancelling terms from the numerator and denominator,

(11)

which can be written in the following form:

This is in the general form of equation (4), where

Thus the parameters from the retrospective QCPG formulation (equation (4)) may be interpreted as follows. The term  is the original genotype effect of genotype  on the prospective scale, divided by the trait variance. (Hence, given parameter estimates  from fitting the QCPG model, the genotype effects on the prospective scale could be obtained by multiplying the estimate of  by the corresponding trait variance, if it were known or estimable.)The terms  correspond to nuisance parameters that allow the model to account for non-Mendelianism and population stratification, as proposed by Kistner and Weinberg [2004]. As discussed in the text, not all of the β′ and α′ are estimable, so restrictions must be made such as setting some of these equal to zero (equivalent to choosing a reference genotype category to which the other genotype effects are compared). This complicates the interpretation of the nuisance parameters. Suppose that all parameters were in fact estimable. Suppose further that there is no non-Mendelianism, so that *P*(*g^*^_i_*∣*g_im_*,*g_if_*)=0.25 for all offspring genotypes consistent with the parental genotypes. In that case, we could write out in full the relationships between the retrospective and prospective parameters implied by equation (11):

In practice, not all the parameters are estimable and we set b01, α′011, α′111 and α′121 (the reference parameters) equal to zero. Assuming that on the prospective scale we also set b150, and rearranging the expressions above, the relationships become

Under the null hypothesis, the prospective genotype effects b0 and b2 equal zero, and so the true values of the retrospective parameters (both the genotype parameters of interest and the nuisance parameters)will also equal zero.

A similar argument applies if, instead of setting b1 and b01 to zero, we set b0 and b00 to zero, so that the genotype parameters are all calculated relative to the homozygous 1/1 genotype. In this case, we still find that under the null hypothesis that b1 and b2 equal zero, the true values of the retrospective parameters (both the genotype parameters of interest and the nuisance parameters) equal zero (data not shown).

The above derivation for the nuisance parameters only works because *P*(*g^*^_i_*∣*g_im_*,*g_if_*)=0.25 in all cases and so is subtracted out when we express α′010, α′110, α′112 and α′122 in terms of α′011, α′111 and α′121. If, in fact, there was non-Mendelianism, we would have instead that

In this case the term cjkl will be nonzero, but fitting α′jkl allows the model to account for this, so that even under the null hypothesis where b0 and b2 equal zero, the nuisance parameters α′jkl will not equal zero.

The effect of population stratification is modelled on the prospective scale via µ =α_m_ + β_gi_ which allows the mean trait value for a child to vary according to the genotype of its parents. If there really were population stratification, the correct model would in fact be μ = μ_*p*_ + Β _*gi*_, where mp denotes the mean trait value in the (unknown) sub-population p fromwhich the child originates. Ifwereplace aMby mp everywhere above, wefind that

Thus the true value of the nuisance parameters should vary according to sub-population, which we do not allow for. However, under the null hypothesis where b0 and b2 equal zero, α′jkl does not vary by subpopulation and so we should still obtain valid inference for the b0 under the null, even though the model misspecification means we may not obtain valid inference under the alternative.

## APPENDIX B

### EXPRESSION OF QPL LIKELIHOOD

We may use a similar argument as in Appendix A to derive the relationship between the α′0 and b00 parameters in the QPL model and the prospective a and b parameters. The only difference is in the summation in the denominator of equation (11), which is over either two or three possible offspring genotype categories corresponding to the categories shown in Table I, (e.g. 1/1, 1/2 (unordered) and 2/2 for offspring of two heterozygous parents, rather than over four genotype categories 1/1, 1/2, 2/1 and 2/2). The result of this is that the relationships implied by equation (11) become

where the terms *P*(*g^*^_i_*∣*g_im_*,*g_if_*)differ according to offspring genotype, for offspring of two heterozygous parents. If, as in Appendix A, we set b1, b01, α′011, α′111 and α′121 to zero, we obtain the following equations:

Under the null hypothesis that β_0_ and β_2_ equal zero, the α″_jkl_ do not all equal zero, and so it is necessary in the QPL model that the α″_jkl_ be included in order to obtain correct inference, even when there is no population stratification or non-Mendelianism.

## APPENDIX C

### DISCUSSION OF NUISANCE PARAMETERS

A difference between the QCPG and QPL methods arises with regard to the number of nuisance parameters estimated. It can be seen in Table I that there are a total of four nuisance parameters, α_010_, α_110_, α_112_, α_122_.However, by fitting the model using a polytomous logistic model as proposed by Kistner and Weinberg, two additional, essentially inestimable, nuisance parameters are estimated. These are referred to in Kistner and Weinberg ([2004] p. 36) as α_012_ and α_120_, corresponding to the situations where either one parent has no copies of the variant allele but the offspring has two copies, or one parent has two copies but the offspring has none. There is no data to estimate these parameters (they correspond to impossible events) but the computer program tries to estimate them since they are included in the model. Implementation of the QPL approach using SAS code available from http://dir.niehs.nih.gov/dirbb/weinbergfiles/qpl.htm (data not shown) indicates that estimation of these two inestimable α′12 and a120 parameters is poor, with very large estimates and confidence intervals, although this does not appear to adversely affect estimation of the six genuinely estimable parameters.

For the QCPG and QPL methods, if there are genotype effects present, the nuisance parameters are non-zero. When population stratification exists, the true values of these nuisance parameters may differ in the different sub-populations (due to differences in α*M* between subpopulations). If estimated in the combined population, the nuisance parameters will be an average of those from the different sub-populations and so will not necessarily provide accurate population-specific estimates. Hence, although the type 1 errors will be correct, we do not necessarily expect the QCPG (or QPL) method to have unbiased parameter estimates when used for estimation of effects under the alternative hypothesis with population stratification, even though they should be unbiased under the null.

When extending the QCPG method to multi-locus haplotypes, the number of nuisance parameters dramatically increases since the number of parental mating types and offspring genotypes increases. The nuisance parameters in the single-locus case are of the form  (equation (11)).

Through simulations (described in the main text), we investigate whether the parental genotype information still required in the nuisance parameters can be captured if the nuisance parameters are replaced by parameters representing the offspring genotype alone (γ*g_i_^*^*) thus reducing the overall number of nuisance parameters. Equation (11) would then become

The hope is that the population stratification can be accounted for by offspring genotype alone, rather than by a combination of offspring and parental genotypes.

## APPENDIX D

### EXPRESSION OF THE QCEPG LIKELIHOOD

The rationale for expressing the likelihood equation (5) in the form of equation (6) is very similar to that used for the QCPG approach. Previously, for the QCPG method, the population mean of the quantitative trait Y (m) was assumed to equal the mean of the prospective QTDT_M_ method such that for an offspring with genotype β_*gi*_, µ= α*_M_* + β_*gi*_, for mating type *M* and offspring genotype effect β_*gi*_. To include maternal genotype or parentof-origin effects, the mean is now assumed to depend on parental mating type and the offspring genotype effect as before, but it now also depends on the maternal genotype effect, β_*gi*_m, and the parent-of-origin effect β*_I__m_*. Therefore,

Hence, the trait distribution can be expressed as a normal distribution with mean m as above, and substituting this into equation (5) gives

where
