# Supplementary material for: Quantitative trait association in parent offspring trios: Extension of case/pseudocontrol method and comparison of prospective and retrospective approaches
Source: Genet Epidemiol. 2007 Jun 4;31(8):813–33. doi: 10.1002/gepi.20243 (PMC2707979; doi:10.1002/gepi.20243)
Supplement: Supplementary file 1 [file gepi0031-0813-SD1.pdf]

Table 1 (**online**). Single locus simulation scenarios

	Models								
	Null	Alt1	Alt2	Popn Strat (Null 1)		Popn Strat (Null 2)		Popn Strat (Alt 1)	
				Popn 1	Popn 2	Popn 1	Popn 2	Popn 1	Popn 2
No. trios per replicate	500	500	500	125	375	125	375	125	375
Minor allele freq.	0.2	0.2	0.2	0.5	0.1	0.1	0.5	0.5	0.1
Mean: 1/1 genotype	2	0	0	2.5	0	2.5	0	2	0
Mean: 1/2 genotype	2	1	0	2.5	0	2.5	0	3	1
Mean: 2/2 genotype	2	2	1	2.5	0	2.5	0	4	2
Standard deviation	2	2	2	1	1	1	1	1	1

Table 2 (**online**). True and estimated means, standard deviations (SD) and coverage (CI) of the 95% confidence intervals for the single locus simulations with one-tail sampling from the upper tail of the offspring trait distribution and no population stratification. The simulation parameters are as shown in Table 1 (**online**).

		Models											
		Null				Alternative 1				Alternative 2			
Method	Parameter	True mean	Mean	(SD)	CI	True mean	Mean	(SD)	CI	True mean	Mean	(SD)	CI
Linear regression	constant	-	4.84	(0.14)	-	-	4.23	(0.25)	-	-	3.38	(0.17)	-
	β_1	0.00	0.00	(0.15)	0.95	1.00	0.15	(0.26)	0.24	0.00	0.01	(0.18)	0.98
	β_2	0.00	0.00	(0.14)	0.96	2.00	0.36	(0.25)	0.00	1.00	0.22	(0.17)	0.01
QTDT_M	β_1	0.00	0.01	(0.18)	0.96	1.00	0.15	(0.29)	0.34	0.00	0.01	(0.21)	0.98
	β_2	0.00	0.01	(0.19)	0.96	2.00	0.36	(0.29)	0.00	1.00	0.23	(0.21)	0.11
QCPG	β'_1	0.00	0.01	(0.23)	0.97	0.25	0.41	(0.84)	0.97	0.00	0.04	(0.32)	0.97
	β'_2	0.00	0.01	(0.24)	0.97	0.50	0.67	(0.84)	0.97	0.25	0.31	(0.32)	0.97
QPL	β''_1	0.00	0.01	(0.23)	0.97	0.25	0.41	(0.84)	0.97	0.00	0.04	(0.32)	0.97
	β''_2	0.00	0.01	(0.24)	0.97	0.50	0.67	(0.84)	0.97	0.25	0.31	(0.32)	0.97
QCPG α's removed	β'_1	0.00	0.00	(0.04)	0.95	0.25	0.24	(0.09)	0.95	0.00	0.01	(0.07)	0.95
	β'_2	0.00	0.00	(0.04)	0.95	0.50	0.41	(0.09)	0.75	0.25	0.22	(0.07)	0.92
QPL α''s removed	β''_1	0.00	0.07	(0.04)	0.61	0.25	0.30	(0.09)	0.96	0.00	0.08	(0.08)	0.85
	β''_2	0.00	0.05	(0.04)	0.79	0.50	0.44	(0.09)	0.87	0.25	0.27	(0.07)	0.95
QCPG α's replaced by g	β'_1	0.00	0.01	(0.22)	0.96	0.25	0.39	(0.82)	0.96	0.00	0.04	(0.31)	0.97
	β'_2	0.00	0.01	(0.23)	0.96	0.50	0.66	(0.82)	0.97	0.25	0.31	(0.31)	0.98
QPL α''s replaced by g	β''_1	0.00	0.01	(0.22)	0.96	0.25	0.36	(0.80)	0.96	0.00	0.01	(0.31)	0.96
	β''_2	0.00	0.01	(0.22)	0.97	0.50	0.63	(0.80)	0.97	0.25	0.28	(0.30)	0.98

Table 3 (**online**). True and estimated means, standard deviations (SD) and coverage (CI) of the 95% confidence intervals for the single locus simulations with one-tail sampling from the upper tail of the offspring trait distribution and with population stratification. The simulation parameters are as shown in Table 1 (**online**).

		Models											
		Popn. Strat. - Null1				Popn. Strat. - Null2				Popn. Strat. - Alt			
Method	Parameter	True mean	Mean	(SD)	CI	True mean	Mean	(SD)	CI	True mean	Mean	(SD)	CI
Linear	constant	-	3.00	(0.04)	-	-	2.49	(0.09)	-	-	3.61	(0.06)	-
regression	β_1	0.00	-0.02	(0.06)	0.92	0.00	0.34	(0.11)	0.23	1.00	0.29	(0.06)	0.00
	β_2	0.00	-0.18	(0.06)	0.16	0.00	0.49	(0.10)	0.01	2.00	0.30	(0.06)	0.00
QTDT_M	β_1	0.00	0.00	(0.08)	0.95	0.00	-0.01	(0.15)	0.98	1.00	0.33	(0.08)	0.00
	β_2	0.00	0.00	(0.10)	0.94	0.00	-0.01	(0.17)	0.97	2.00	0.62	(0.10)	0.00
QCPG	β'_1	0.00	0.00	(0.14)	0.95	0.00	-0.01	(0.48)	0.96	0.25	1.33	(0.45)	0.33
	β'_2	0.00	0.01	(0.18)	0.94	0.00	0.00	(0.49)	0.95	0.50	1.97	(0.46)	0.07
QPL	β''_1	0.00	0.00	(0.14)	0.95	0.00	-0.01	(0.48)	0.96	0.25	1.33	(0.45)	0.33
	β''_2	0.00	0.01	(0.18)	0.94	0.00	0.00	(0.49)	0.95	0.50	1.97	(0.46)	0.07
QCPG	β'_1	0.00	0.00	(0.03)	0.94	0.00	0.00	(0.09)	0.95	0.25	0.31	(0.05)	0.87
α's removed	β'_2	0.00	0.00	(0.04)	0.95	0.00	0.00	(0.10)	0.95	0.50	0.51	(0.05)	0.97
QPL	β''_1	0.00	0.07	(0.03)	0.40	0.00	0.10	(0.09)	0.83	0.25	0.35	(0.05)	0.52
α''s removed	β''_2	0.00	0.00	(0.04)	0.96	0.00	0.08	(0.09)	0.89	0.50	0.50	(0.05)	0.97
QCPG α's	β'_1	0.00	0.00	(0.14)	0.95	0.00	0.00	(0.45)	0.95	0.25	1.33	(0.45)	0.34
replaced by g	β'_2	0.00	0.01	(0.18)	0.94	0.00	0.01	(0.47)	0.95	0.50	1.92	(0.46)	0.09
QPL α''s	β''_1	0.00	0.00	(0.14)	0.95	0.00	0.08	(0.44)	0.96	0.25	1.30	(0.44)	0.36
replaced by g	β''_2	0.00	-0.01	(0.16)	0.96	0.00	0.12	(0.45)	0.96	0.50	1.84	(0.44)	0.10

Table 4 (**online**). True and estimated means, standard deviations (SD) and coverage (CI) of the 95% confidence intervals for the single locus simulations with two-tailed sampling from the upper and lower tails of the offspring trait distribution and no population stratification. The simulation parameters are as shown in Table 1 (**online**).

		Models											
		Null				Alternative 1				Alternative 2			
Method	Parameter	True mean	Mean	(SD)	CI	True mean	Mean	(SD)	CI	True mean	Mean	(SD)	CI
Linear regression	constant	-	1.95	(0.93)	-	-	-2.76	(0.28)	-	-	-1.96	(0.76)	-
	β_1	0.00	0.03	(1.03)	0.95	1.00	1.87	(0.38)	0.91	0.00	0.00	(0.83)	0.97
	β_2	0.00	0.02	(0.97)	0.96	2.00	6.07	(0.34)	0.00	1.00	4.10	(0.80)	0.06
QTDT_M	β_1	0.00	0.02	(1.23)	0.95	1.00	1.85	(0.68)	0.91	0.00	-0.02	(1.03)	0.97
	β_2	0.00	-0.01	(1.26)	0.96	2.00	6.04	(0.77)	0.01	1.00	4.08	(1.08)	0.24
QCPG	β_1'	0.00	0.00	(0.07)	0.96	0.25	0.33	(0.20)	0.97	0.00	0.01	(0.10)	0.97
	β_2'	0.00	0.00	(0.08)	0.96	0.50	0.57	(0.20)	0.97	0.25	0.25	(0.10)	0.96
QPL	β_1''	0.00	0.00	(0.07)	0.96	0.25	0.33	(0.20)	0.97	0.00	0.01	(0.10)	0.97
	β_2''	0.00	0.00	(0.08)	0.96	0.50	0.57	(0.20)	0.97	0.25	0.25	(0.10)	0.96
QCPG α's removed	β_1'	0.00	0.01	(0.06)	0.95	0.25	0.29	(0.08)	0.94	0.00	0.00	(0.07)	0.95
	β_2'	0.00	0.01	(0.06)	0.96	0.50	0.51	(0.08)	0.96	0.25	0.24	(0.07)	0.96
QPL α''s removed	β_1''	0.00	0.03	(0.06)	0.93	0.25	0.19	(0.07)	0.89	0.00	-0.06	(0.06)	0.87
	β_2''	0.00	0.02	(0.06)	0.96	0.50	0.42	(0.07)	0.83	0.25	0.18	(0.06)	0.86
QCPG α's replaced by g	β_1'	0.00	0.00	(0.07)	0.96	0.25	0.32	(0.19)	0.98	0.00	0.01	(0.09)	0.97
	β_2'	0.00	0.00	(0.07)	0.97	0.50	0.56	(0.19)	0.98	0.25	0.25	(0.09)	0.96
QPL α''s replaced by g	β_1''	0.00	0.00	(0.07)	0.96	0.25	0.30	(0.19)	0.96	0.00	-0.01	(0.09)	0.95
	β_2''	0.00	0.00	(0.07)	0.97	0.50	0.54	(0.19)	0.96	0.25	0.23	(0.09)	0.94

Table 5 (**online**). True and estimated means, standard deviations (SD) and coverage (CI) of the 95% confidence intervals for the single locus simulations with two-tailed sampling from the upper and lower tails of the offspring trait distribution and with population stratification. The simulation parameters are as shown in Table 1 (**online**).

		Models											
		Popn. Strat. - Null1				Popn. Strat. - Null2				Popn. Strat. - Alt			
Method	Parameter	True mean	Mean	(SD)	CI	True mean	Mean	(SD)	CI	True mean	Mean	(SD)	CI
Linear regression	constant	-	3.72	(0.16)	-	-	-1.73	(0.15)	-	-	0.04	(0.27)	-
	β_1	0.00	-1.34	(0.25)	0.01	0.00	1.32	(0.23)	0.01	1.00	1.43	(0.33)	0.93
	β_2	0.00	-4.30	(0.21)	0.00	0.00	4.28	(0.21)	0.00	2.00	2.99	(0.30)	0.36
QTDT_M	β_1	0.00	-0.01	(0.29)	1.00	0.00	-0.02	(0.27)	1.00	1.00	2.99	(0.37)	0.01
	β_2	0.00	-0.01	(0.47)	0.95	0.00	0.00	(0.47)	0.95	2.00	6.19	(0.37)	0.00
QCPG	β'_1	0.00	-0.03	(0.20)	0.96	0.00	0.02	(0.18)	0.96	0.25	0.83	(0.35)	0.05
	β'_2	0.00	-0.04	(0.20)	0.96	0.00	0.02	(0.19)	0.97	0.50	1.45	(0.36)	0.00
QPL	β''_1	0.00	-0.03	(0.20)	0.96	0.00	0.02	(0.18)	0.96	0.25	0.83	(0.35)	0.05
	β''_2	0.00	-0.04	(0.20)	0.96	0.00	0.02	(0.19)	0.97	0.50	1.45	(0.36)	0.00
QCPG α's removed	β'_1	0.00	0.00	(0.05)	0.95	0.00	0.00	(0.08)	0.96	0.25	0.68	(0.38)	0.33
	β'_2	0.00	0.00	(0.06)	0.95	0.00	0.00	(0.10)	0.95	0.50	0.99	(0.38)	0.14
QPL α''s removed	β''_1	0.00	0.05	(0.05)	0.80	0.00	-0.06	(0.08)	0.91	0.25	0.63	(0.30)	0.27
	β''_2	0.00	0.01	(0.06)	0.95	0.00	-0.04	(0.09)	0.94	0.50	0.89	(0.30)	0.28
QCPG α's replaced by g	β'_1	0.00	-0.04	(0.19)	0.96	0.00	0.02	(0.18)	0.97	0.25	0.70	(0.19)	0.02
	β'_2	0.00	-0.04	(0.20)	0.96	0.00	0.02	(0.18)	0.97	0.50	1.31	(0.20)	0.00
QPL α''s replaced by g	β''_1	0.00	-0.06	(0.18)	0.97	0.00	0.04	(0.17)	0.98	0.25	0.64	(0.19)	0.11
	β''_2	0.00	-0.09	(0.19)	0.97	0.00	0.07	(0.18)	0.98	0.50	1.20	(0.19)	0.00

Table 6 (**online**). Powers and type I errors for the single locus simulations for the six scenarios in Table 1 (**online**) under the three sampling schemes. Methods in which the nuisance parameters have been removed from the model are indicated by 'No α', and 'g' denotes a model in which the nuisance parameters have been replaced by the offspring genotype.

		Models																	
		Null			Alternative 1			Alternative 2			Strat Null			Strat Null2			Strat Alt		
		p value			p value			p value			p value			p value			p value		
Selection	Method	0.05	0.01	0.001[a]	0.05	0.01	0.001[a]	0.05	0.01	0.001[a]	0.05	0.01	0.001[a]	0.05	0.01	0.001[a]	0.05	0.01	0.001[a]
Random	Regression	0.045	0.010	0.002	0.999	0.996	0.991	1.000	0.987	0.936	1.000	1.000	1.000	1.000	1.000	1.000	0.377	0.162	0.039
	QTDT_M	0.046	0.011	0.001	0.975	0.911	0.761	0.927	0.796	0.520	0.071	0.016	0.004	0.038	0.003	0.000	1.000	1.000	0.997
	QCPG	0.039	0.007	0.000	0.972	0.895	0.674	0.922	0.764	0.456	0.036	0.007	0.000	0.041	0.007	0.000	1.000	0.998	0.985
	QPL	0.039	0.007	0.000	0.972	0.895	0.674	0.922	0.764	0.456	0.036	0.007	0.000	0.041	0.007	0.000	1.000	0.998	0.985
	QCPG (No α)	0.044	0.008	0.000	0.896	0.720	0.408	0.903	0.736	0.412	0.035	0.009	0.000	0.043	0.009	0.000	0.771	0.536	0.234
	QPL (No α)	0.109	0.023	0.004	0.884	0.676	0.334	0.883	0.681	0.370	0.093	0.015	0.004	0.030	0.007	0.001	0.769	0.462	0.148
	QCPG (g)	0.043	0.008	0.000	0.974	0.896	0.675	0.926	0.768	0.455	0.037	0.006	0.001	0.045	0.007	0.001	1.000	0.998	0.984
	QPL (g)	0.036	0.008	0.000	0.979	0.900	0.687	0.926	0.776	0.459	0.052	0.010	0.001	0.044	0.006	0.000	0.999	0.996	0.951
Top	Regression	0.045	0.007	0.002	0.801	0.528	0.217	0.790	0.517	0.205	0.880	0.697	0.424	0.993	0.965	0.845	0.851	0.530	0.130
	QTDT_M	0.050	0.007	0.001	0.511	0.251	0.070	0.500	0.231	0.066	0.060	0.012	0.001	0.035	0.006	0.001	0.999	0.998	0.985
	QCPG	0.043	0.005	0.001	0.537	0.247	0.066	0.516	0.237	0.064	0.048	0.010	0.001	0.056	0.006	0.001	0.999	0.996	0.957
	QPL	0.043	0.005	0.001	0.537	0.247	0.066	0.516	0.237	0.064	0.048	0.010	0.001	0.056	0.006	0.001	0.999	0.996	0.957
	QCPG (No α)	0.049	0.009	0.002	1.000	1.000	1.000	1.000	1.000	1.000	0.054	0.008	0.000	0.063	0.012	0.001	1.000	1.000	1.000
	QPL (No α)	0.354	0.143	0.049	1.000	1.000	1.000	1.000	1.000	1.000	0.727	0.486	0.243	0.155	0.041	0.007	1.000	1.000	1.000
	QCPG (g)	0.044	0.004	0.001	0.542	0.258	0.065	0.517	0.240	0.064	0.049	0.011	0.001	0.047	0.008	0.001	1.000	0.994	0.952
	QPL (g)	0.038	0.004	0.001	0.543	0.262	0.065	0.515	0.243	0.060	0.043	0.012	0.001	0.053	0.009	0.002	0.999	0.992	0.927
Top+	Regression	0.048	0.006	0.000	1.000	1.000	1.000	1.000	1.000	0.997	1.000	1.000	1.000	1.000	1.000	1.000	1.000	1.000	0.480
Bottom	QTDT_M	0.035	0.006	0.000	1.000	1.000	0.654	1.000	1.000	0.370	0.105	0.030	0.000	0.095	0.032	0.000	1.000	1.000	1.000
	QCPG	0.032	0.006	0.000	1.000	1.000	0.067	1.000	1.000	0.023	0.044	0.007	0.000	0.045	0.003	0.000	1.000	1.000	0.999
	QPL	0.032	0.006	0.000	1.000	1.000	0.067	1.000	1.000	0.023	0.044	0.007	0.000	0.045	0.003	0.000	1.000	1.000	0.999
	QCPG (No α)	0.034	0.006	0.000	1.000	1.000	0.236	1.000	1.000	0.036	0.061	0.015	0.000	0.045	0.007	0.000	1.000	1.000	0.680
	QPL (No α)	0.057	0.009	0.000	1.000	1.000	0.068	1.000	1.000	0.043	0.208	0.077	0.000	0.096	0.028	0.000	1.000	1.000	0.115
	QCPG (g)	0.035	0.006	0.000	1.000	1.000	0.085	1.000	1.000	0.033	0.046	0.009	0.000	0.042	0.008	0.000	1.000	1.000	1.000
	QPL (g)	0.035	0.004	0.000	1.000	1.000	0.076	1.000	1.000	0.033	0.084	0.019	0.000	0.071	0.019	0.000	1.000	1.000	1.000

[a]Except for Top+Bottom sampling scheme, in which case results are shown for a more stringent p value of 10^{-15}

Table 7 (**online**). Two-locus haplotype simulation scenarios

	Models					
	Null	Alt	Popn. Strat. - Null		Popn. Strat. - Alt	
			Popn. 1	Popn. 2	Popn. 1	Popn. 2
No. trios per replicate	1000	1000	250	750	250	750
Frequency 1-1	0.6	0.6	0.6	0.1	0.6	0.1
Frequency 1-2	0.1	0.1	0.1	0.15	0.1	0.15
Frequency 2-1	0.15	0.15	0.15	0.1	0.15	0.1
Frequency 2-2	0.15	0.15	0.15	0.6	0.15	0.6
Mean 1-1	0	0	0	5	0	5
Mean 1-2	0	1	0	5	1	6
Mean 2-1	0	2	0	5	2	7
Mean 2-2	0	3	0	5	3	8
Standard Deviation	1	1	1	1	1	1

Table 8 (**online**). Results comparing extensions of the QCEPG, $QTDT_M$ and QPL methods to include maternal genotype and parent-of-origin effects under the null.

		Maternal genotype			Parent-of-origin		
Method	Parameter	True mean	Mean	(SD)	True mean	Mean	(SD)
QCEPG	β_1	1	1.113	0.422	1	1.027	0.418
	β_2	2	1.960	0.430	2	1.980	0.430
	β_{m1}	0	-0.021	0.262	-	-	-
	β_{m2}	0	0.010	0.257	-	-	-
	β_I	-	-	-	0	0.002	0.123
QPL	β_0	-1	-0.982	0.530	-1	-0.982	0.530
	β_2	1	0.941	0.475	1	0.941	0.475
	δ_{01}	0	-0.007	0.255	-	-	-
	δ_{12}	0	0.000	0.088	-	-	-
	λ_1	-	-	-	0	-0.003	0.101
$QTDT_M$	β_1	1	0.998	0.247	1	0.998	0.256
	β_2	2	1.998	0.244	2	1.998	0.278
	β_{m1}	0	0.001	0.248	-	-	-
	β_{m2}	0	0.000	0.245	-	-	-
	β_I	-	-	-	0	-0.001	0.129

Table 9 (**online**). Results comparing extensions of the QCEPG, $QTDT_M$ and QPL methods to include maternal genotype and parent-of-origin effects under the alternative of maternal genotype or parent-of-origin effects (or both).

		Maternal genotype			Parent-of-origin			Maternal genotype and parent-of-origin		
Method	Parameter	True mean	Mean	(SD)	True mean	Mean	(SD)	True mean	Mean	(SD)
QCEPG	β_1	1	1.134	0.431	1	1.176	0.757	1	1.294	0.646
	β_2	2	1.988	0.458	2	2.129	0.775	2	2.228	0.687
	β_{m1}	0.5	0.536	0.258	-	-	-	0.5	0.706	0.450
	β_{m2}	1	0.982	0.272	-	-	-	1	1.232	0.536
	β_I	-	-	-	1	0.967	0.142	1	0.705	0.596
QPL	β_0	-1	-0.917	0.623	-1	-1.212	0.813	-1	-1.171	0.748
	β_2	1	0.823	0.596	1	1.166	0.809	1	1.006	0.736
	δ_{01}	0.5	0.540	0.259	-	-	-	0.5	0.491	0.367
	δ_{12}	0.5	0.416	0.098	-	-	-	0.5	0.779	0.098
	λ_1	-	-	-	1	0.483	0.082	1.000	0.550	0.081
$QTDT_M$	β_1	1	1.030	0.277	1	1.029	0.283	1	1.028	0.290
	β_2	2	2.032	0.262	2	2.028	0.290	2	2.026	0.333
	β_{m1}	0.5	0.485	0.237	-	-	-	0.5	0.483	0.263
	β_{m2}	1	0.992	0.245	-	-	-	1	0.986	0.317
	β_I	-	-	-	1	1.005	0.138	1	1.006	0.207

Table 10 (**online**). Powers and Type I errors when estimating maternal genotype and/or parent-of-origin effect

	Type I Error						Power											
	Maternal genotype			Parent-of-origin			Maternal genotype			Parent-of-origin			Maternal genotype given parent-of-origin			Parent-of-origin given maternal genotype		
	p value			p value			p value			p value			p value			p value		
Method	0.05	0.01	0.001	0.05	0.01	0.001	0.05	0.01	0.001	0.05	0.01	0.001	0.05	0.01	0.001	0.05	0.01	0.001
QCEPG	0.133	0.058	0.009	0.059	0.011	0.000	1.000	1.000	0.990	1.000	1.000	1.000	0.949	0.808	0.545	0.990	0.949	0.848
QPL	0.058	0.009	0.001	0.040	0.005	0.000	1.000	1.000	0.976	1.000	1.000	0.988	1.000	1.000	1.000	1.000	1.000	1.000
QTDT_{M}	0.052	0.012	0.003	0.060	0.011	0.000	1.000	1.000	1.000	1.000	1.000	1.000	0.930	0.850	0.600	1.000	1.000	0.960
